# ADAM10 Cell Surface Expression but Not Activity Is Critical for *Staphylococcus aureus* α-Hemolysin-Mediated Activation of the NLRP3 Inflammasome in Human Monocytes

**DOI:** 10.3390/toxins8040095

**Published:** 2016-03-30

**Authors:** Ejiofor A.D. Ezekwe, Chengyu Weng, Joseph A. Duncan

**Affiliations:** 1 Department of Pharmacology, School of Medicine, University of North Carolina at Chapel Hill, Chapel Hill, NC 27599, USA; cweng@live.unc.edu (C.W.); joseph_duncan@med.unc.edu (J.A.D.); 2 Department of Medicine/Division of Infectious Diseases, School of Medicine, University of North Carolina at Chapel Hill, Chapel Hill, NC 27599, USA

**Keywords:** NLRP3, ADAM10, α-hemolysin

## Abstract

The *Staphylococcus aureus* toxin, α-hemolysin, is an important and well-studied virulence factor in staphylococcal infection. It is a soluble monomeric protein that, once secreted by the bacterium, forms a heptameric pore in the membrane of a broad range of host cell types. Hemolysin was recently discovered to bind and activate a disintegrin and metalloprotease 10 (ADAM10). In epithelial and endothelial cells, ADAM10 activation is required for the toxin’s activity against these cells. In host monocytic cells, α-hemolysin activates the nucleotide-binding domain and leucine-rich repeat containing gene family, pyrin domain containing 3 (NLRP3) inflammasome leading to production of pro-inflammatory cytokines and cell death. We now show that ADAM10 is critical for α-hemolysin-mediated activation of the NLRP3 inflammasome in human monocytes as siRNA knockdown or chemical blockade of ADAM10-α-hemolysin interaction leads to diminished inflammasome activation and cell death by reducing the available ADAM10 on the cell surface. Unlike epithelial cell and endothelial cell damage, which requires α-hemolysin induced ADAM10 activation, ADAM10 protease activity was not required for NLRP3 inflammasome activation. This work confirms the importance of ADAM10 in immune activation by α-hemolysin, but indicates that host cell signal induction by the toxin is different between host cell types.

## 1. Introduction

*Staphylococcus aureus* is a gram-positive bacterium that is responsible for causing infections that lead to severe morbidity and mortality. *S. aureus* causes infections in a broad range of host tissues including the skin, vascular, and respiratory systems [1]. It is also a growing public health concern because of the emergence of antibiotic resistance including methicillin resistant strains that cause both hospital and community acquired infections [2,3,4].

*Staphylococcus aureus* produces an array of virulence factors that are important for the pathogenesis of infections caused by these bacteria. Among these virulence factors are several pore-forming toxins that attack host cells by permeabilizing their cell membranes. The pore-forming toxin, α-hemolysin (Hla) is one of the best studied of these factors and is critical for virulence in mouse models of infections caused by *S. aureus* [5,6,7,8]. Hla is active against cells from a variety of tissues including respiratory epithelium, endothelium, immune cells, and keratinocytes [8]. This broad range of cellular targets stems from the nearly universal expression of the host cellular receptor for Hla, A Disintegrin and Metalloproteinase-10 or ADAM10 [9]. Additionally, the level of ADAM10 expression on a given cell type dictates sensitivity to the toxin [9]. Genetic loss or chemical inhibition of ADAM10 protects cellular targets from Hla in tissue culture and mitigates Hla-induced pathology in mice [9,10,11,12,13,14]. Further, mice treated with ADAM10 inhibitors or with tissue specific knock out of ADAM10 exhibit resistance to *S. aureus* infection. In epithelial and endothelial cells, Hla’s interaction with ADAM10 leads to the activation of ADAM10’s metalloproteinase activity. This enhanced protease activity leads to the cleavage of cell surface adhesins, like E-cadherin, and disruption of cell-to-cell contacts [9,12]. Consequently, it is believed that activation of ADAM10 by Hla is important for *S. aureus* ability to penetrate epithelial and endothelial barriers and thus cause invasive infection.

Hla is also a potent activator of the innate immune signaling protein, Nucleotide-binding domain and Leucine-Rich repeat containing family Pyrin domain containing 3 (NLRP3) inflammasome [15,16]. The active NLRP3 inflammasome is a protein complex containing NLRP3 and the apoptosis-associated speck-like protein containing a caspase recruitment domain (ASC) which is responsible for activation of the cysteine proteinase caspase-1. Active caspase-1 then goes on to proteolytically process the cytosolic, pro-inflammatory cytokines pro-IL-1β and pro-IL-18 into their active, secreted forms [17,18]. In addition, NLRP3 activation leads to a program of necrotic cell death termed pyroptosis [18,19,20]. Mice with genetic deletion of *Nlrp3* have diminished inflammation in Hla-induced pneumonitis models and decreased severity of infection in a mouse model of Staphylococcal pneumonia [21]. Conversely, in murine models of *S. aureus* skin infection IL-1β production is important for proper bacterial clearance [13,22]. In this study, we sought to determine the role of Hla induced ADAM10 activation in the NLRP3 inflammasome signaling pathway. We show that in human monocytes ADAM10 mediates NLRP3 activation and that the level of ADAM10 cell surface expression and not its protease activity, is important for NLRP3 activation.

## 2. Results and Discussion

### 2.1. ADAM10 Expression Is Required for α-Hemolysin Induced Cell Death in Human Monocyte-Derived Cells

**Figure 1:**
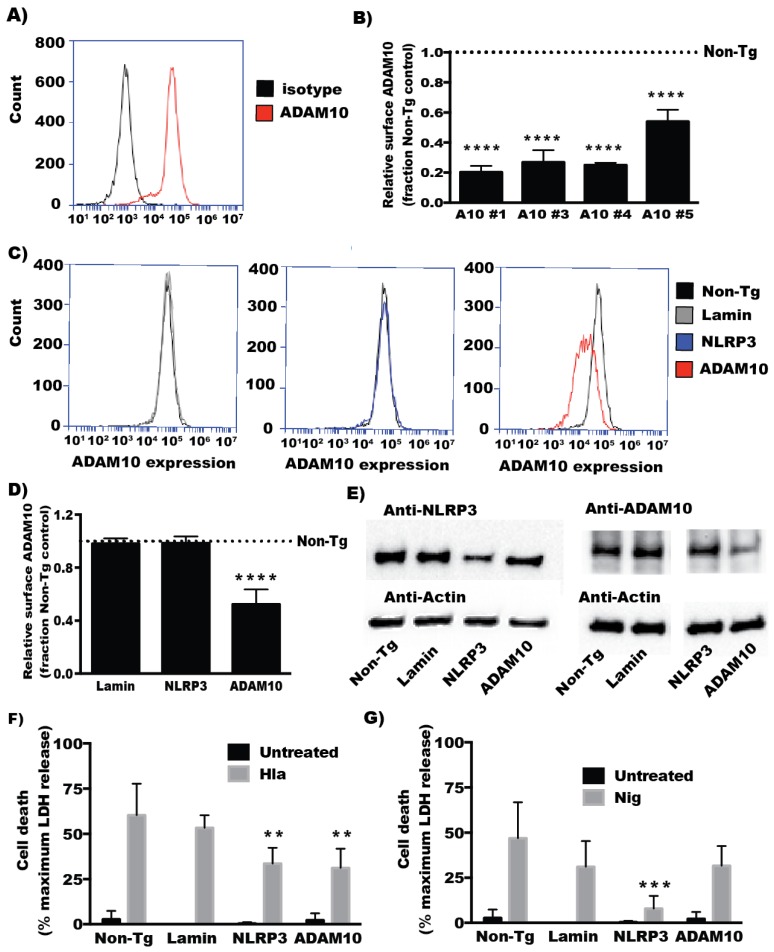
(**A**) Histogram showing staining with ADAM10-directed antibody compared to isotype control measured by flow cytometry. (**B**–**D**) THP1 cells were transfected with the indicated siRNA three days prior to assessing ADAM10 cell surface expression via flow cytometry, as detailed in the experimental methods section and demonstrated in (**A**). (**B**) The relative surface expressed ADAM10 mean fluorescence intensity of THP1 cells transfected with 4 different siRNA targeting ADAM10 as compared to the non-targeting control siRNA transfected cells; (**C**) Histogram showing fluorescence intensity of ADAM10 staining between cells transfected with non-targeting siRNA and siRNA pools targeting Lamin, NLRP3, and ADAM10; (**D**) The relative surface expressed ADAM10 mean fluorescence intensity of siRNA pool transfected THP1 cells as compared to the non-targeting control. For both (**B**,**D**), *n* = 3; (**E**) Whole cell lysates from cells transfected with the indicated siRNA pools were analyzed by immunoblot analysis with antibodies for ADAM10, NLRP3, and Actin as a loading control; (**F**,**G**) siRNA-transfected THP1 cells were treated with α-hemolysin (Hla) or Nigericin (Nig) for 1 h. Culture supernatants from untreated and toxin-treated cells were assayed for LDH production as compared to a detergent-lysis control (*n* = 3); For (**B**,**D**), **** indicates statistically significant difference from Non-Tg transfected cells (*p* ≤ 0.0001) determined by one-way ANOVA with Dunnett’s multiple comparisons testing; For (**F**,**G**), ** and *** indicates statistically significant difference from Non-Tg transfected cells (*p* ≤ 0.01 and *p* ≤ 0.001 respectively) determined by two-way ANOVA testing with Dunnett’s multiple comparison testing.

Previous work has shown ADAM10 to be important for the activity of α-hemolysin (Hla) towards a variety of host cell types [9,11,12]. Loss of expression of ADAM10 using either siRNA in immortalized human epithelial cells or tissue specific genetic knock-out in mouse epithelial cells blocks Hla induced cell death [9]. Lung epithelium specific knock out of the ADAM10 gene protects mice from pulmonary injury induced by Hla inhalation or live *S. aureus* instillation [10]. Targeted deletion of ADAM10 in mouse myeloid cells also protects them from Hla induced death in a murine pneumonia model [13]. We sought to confirm that ADAM10 expression is required for human monocytic cell responsiveness to Hla. Monocytic THP1 cells were transfected with siRNA directed against ADAM10 (both individual siRNAs and pooled siRNA) and after three days cell surface expression was characterized by flow cytometry. We were able to achieve significant reductions in detectable cell surface expression of ADAM10 as compared to our non-targeting siRNA controls (Figure 1B–D). Immunoblot analysis also showed reductions of total ADAM10 (Figure 1E). Because it has previously been shown that NLRP3 expression is required for Hla-induced cell death in monocytes, siRNA directed to NLRP3 were used as a positive control. While siRNA directed to NLRP3 reduced NLRP3 expression levels, it had no effect on ADAM10 levels measured by flow cytometry or immunoblot (Figure 1C–E). siRNA transfected THP1 cells were then treated with Hla and cytolysis was subsequently assessed by measuring release of cytosolic LDH into the culture supernatant. Depletion of NLRP3 or ADAM10 by siRNA significantly reduced Hla-induced LDH release as compared with controls (Figure 1F). To demonstrate the effect of ADAM10 depletion was specific to Hla-induced cell death and not generally suppressive of NLRP3 activation, siRNA transfected cells were also treated with nigericin, a pore forming toxin known to activate NLRP3, and assessed for cell death [23]. As expected, NLRP3 depletion blocked nigericin-induced death while ADAM10 depletion had no effect (Figure 1G).

### 2.2. ADAM10 Expression Is Required for Hla-Mediated NLRP3 Inflammasome Activation in Monocytes

**Figure 2:**
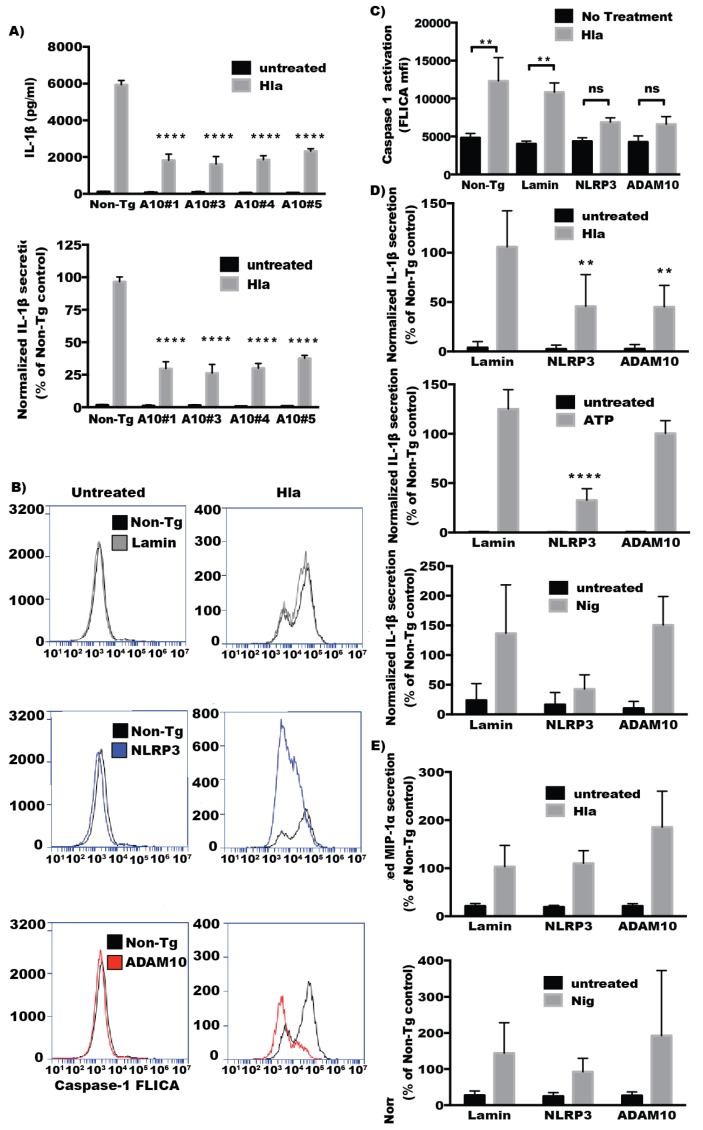
THP1 cells were treated with the indicated siRNA three days prior to assessment, as in Figure 1. siRNA-transfected THP1 cells were treated with LPS (100 ng/mL) for 3 h before the addition of an NLRP3 simulating agent for 1 h. Cell culture supernatants were then assayed for cytokine production. For some analysis the relative secretion of cytokine as compared with toxin-treated, non-targeting siRNA-transfected control cells is plotted. (**A**) absolute (**top**) and relative (**bottom**) IL-1β secretion in hemolysin treated THP1 cells transfected with individual ADAM10 siRNAs (*n* = 3). (**B**) THP1 cells transfected with the indicated siRNA pool were treated with Caspase-1 FLICA reagent prior to the addition of α-hemolysin for 30 min. Cells are then washed, fixed and assayed by flow cytometry. Representative histograms showing caspase-1 FLICA reagent based fluorescence in resting (**left**) and α-hemolysin-treated (**right**) cells previously transfected with the indicated siRNA; (**C**) Caspase-1 FLICA activation mean fluorescence intensity in cells treated with hemolysin (*n* = 3); (**D**) Relative IL-1β secretion as compared with non-targeting control in cells treated with α-hemolysin (*n* = 7), **top**, ATP (*n* = 4), **middle**, and nigericin (*n* = 3), **bottom**; (**E**) Relative MIP1-α secretion as compared with non-targeting control in cells treated with hemolysin (*n* = 3) or nigericin (*n* = 3); For (**A**,**D**) ** and **** indicate statistically significant difference from Non-Tg (*p* ≤ 0.01 and *p* ≤ 0.0001, respectively) determined by one-way ANOVA with Dunnett’s multiple comparisons testing; For (**C**) ** Indicates statistically significant difference between no treatment and Hla treatment (*p* ≤ 0.01) determined by one-way ANOVA with Sidak’s multiple comparision testing.

Hla-induces inflammatory cell death by activating the NLRP3 inflammasome in monocytes [15]. Activation of the NLRP3 inflammasome leads to activation of the cysteine proteinase caspase-1 and subsequent processing and secretion of the cytokines IL-1β and IL-18 [17]. To determine whether ADAM10 is required for Hla-mediated NLRP3 activation, THP1 cells transfected with individual siRNA directed against ADAM10 as well as pooled siRNA targeting ADAM10 and NLRP3 were challenged with α-hemolysin and IL-1β secretion by the cells as well as activation of caspase-1 were assessed. Cells depleted of ADAM10 expression using four different siRNA against ADAM10 all demonstrated a marked reduction in IL-1β secretion when compared to non-targeting control siRNA (Figure 2A). Caspase-1 activation was assessed by accumulation of a fluorescent inhibitor of caspase-1 (caspase-1 FLICA) after toxin administration. Knockdown of ADAM10 or NLRP3 by pooled siRNA transfection of THP1 cells significantly decreased caspase-1 activation in response to Hla (Figure 2B,C). Cells depleted of ADAM10 or NLRP3 expression by transfection of pooled siRNA exhibited markedly reduced release of IL-1β in response to Hla exposure (Figure 2D). To test whether ADAM10 depletion effected NLRP3 activation in general or only in response to Hla, siRNA-transfected cells were treated with nigericin or ATP, which activates NLRP3 through activation of the purogenic P2X7 receptor [24]. ADAM10 knockdown did not affect secretion of IL-1β after treatment with either ATP or nigericin (Figure 2D). In contrast to the reductions in Hla-induced IL-1β secretion observed after transfection with NLRP3 or ADAM10 directed siRNA, secretion of MIP-1α, a cytokine that is not dependent on caspase-1 proteolysis for secretion, was not reduced at all in these cells relative to the control siRNA transfected cells (Figure 2E). Thus, ADAM10 expression is critical for NLRP3 activation by Hla but not by other NLRP3 activating stimuli.

### 2.3. The Protease Activity of ADAM10 Is Not Required for Hla-Mediated Activation of NLRP3-Induced Cell Death

**Figure 3:**
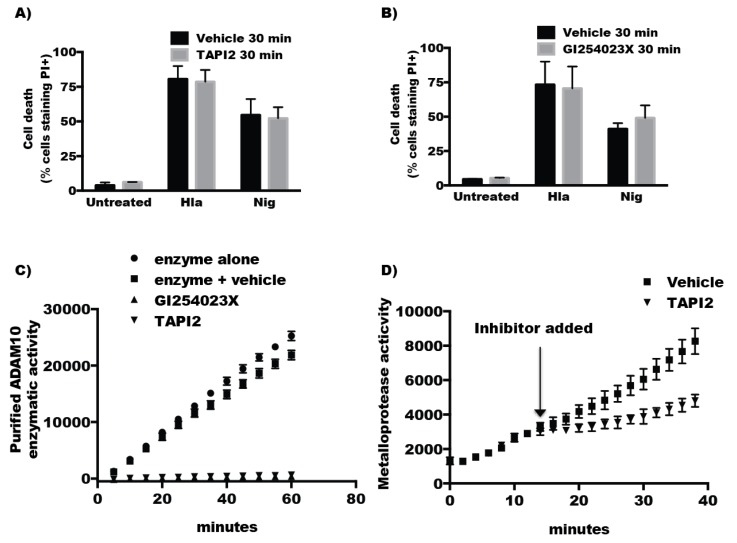
(**A**,**B**) THP1 cells were incubated with either GI254023X or TAPI2 inhibitors or inhibitor vehicle (DMSO for GI254023X or H_2_O for TAPI2) for 30 min, propidium iodide (PI) was then added to the cells followed by treatment with either nothing (untreated, *n* = 3), α-hemolysin (Hla, *n* = 3), or nigericin (Nig, *n* = 3) for 1 h. Cell death was assessed by measuring cells that stained positive for PI using flow cytometry. (**C**) Purified recombinant ADAM10 protein was mixed with vehicle (square), 20 μM GI254023X (upward triangle) or 100 μM TAPI2 (downward triangle) and immediately assayed for ADAM10 protease activity as described in the Materials and Methods for 60 min with measurements taken every five minutes; (**D**) Metalloprotease activity was measured in THP1 cells by incubating intact cells with a fluorogenic peptide substrate and measuring fluorescent intensity every 2 min. After 10 min, the metalloproteinase inhibitor TAPI2 (100 μM) or vehicle was added to the reaction and fluorescence intensity was measured an additional 30 min.

Chemical inhibition of ADAM10 using GI254023X, a specific inhibitor of ADAM10 reduces Hla-mediated cytotoxicity and cleavage of extracellular E-cadherin in epithelial and endothelial cells [10,12,25]. To determine whether the protease activity of ADAM10 was required for Hla-induced NLRP3 activation, we treated cells with TAPI2, a non-specific metalloprotease inhibitor or GI254023X. THP1 cells were treated with inhibitor for 15–30 min prior to challenge with Hla. Short-term treatment of THP1 cells (15–30 min) with these inhibitors did not reduce Hla-induced cytotoxicity (Figure 3A,B). Inoshima *et al.* demonstrated that treatment of epithelial cells with GI254023X led to diminished ADAM10-mediated E-cadherin cleavage within minutes of addition of the inhibitor [10]. To ensure short-term treatment with ADAM10 inhibitors could suppress protease activity in the 30 min time-frame of our experiments, we tested the effect of the inhibitors on measurable protease activity. We found that TAPI2 and GI254023X were able to immediately impact the rate of peptide substrate cleavage by purified ADAM10 (Figure 3C). Further, total metalloproteinase activity in intact THP1 cells was immediately diminished by the addition of TAPI2 (Figure 3D). Combined these results demonstrate that the proteinase activity of ADAM10 is not required α-hemolysin-induced cell death.

### 2.4. Inhibitors of ADAM10 Protease Activity Reduce Hla-Mediated Activation of the NLRP3 Inflammasome through down Regulation of Surface ADAM10 Levels

**Figure 4:**
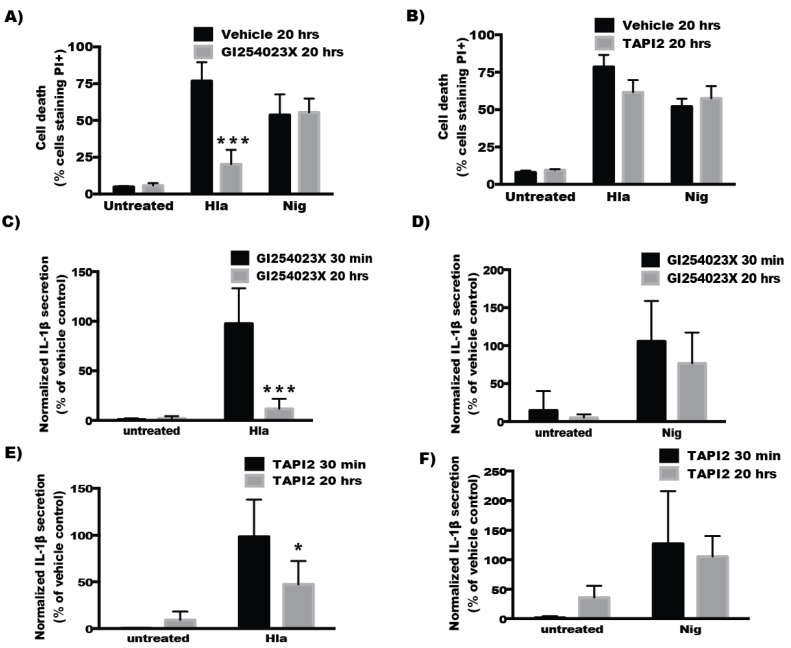
(**A**,**B**) THP1 cells were incubated with either GI254023X or TAPI2 inhibitors or inhibitor vehicle (DMSO for GI254023X or H_2_O for TAPI2) for 20 h, propidium iodide (PI) was then added to the cells followed by treatment with either nothing (untreated, *n* = 3), α-hemolysin (Hla, *n* = 3), or nigericin (Nig, *n* = 3) for 1 h. (**C**–**F**) THP1 cells were incubated with the indicated inhibitors either overnight (20 h) or for 30 min. Cells were subsequently incubated with LPS for 3 h followed by no addition (untreated, *n* = 3), α-hemolysin (Hla, *n* = 3), or nigericin (Nig, *n* = 3) for one hour. Cell-culture supernatants were then collected and assayed for IL-1β. Results are reported as either relative secretion to secretion from vehicle exposed cells subsequently treated with the indicated toxin (Hla or Nig). * and *** indicates statistically significant difference from vehicle treated cells intoxicated with Hla (*p* ≤ 0.05 or *p* ≤ 0.001, respectively) determined by one-way ANOVA with Dunnett’s multiple comparisons testing.

**Figure 5:**
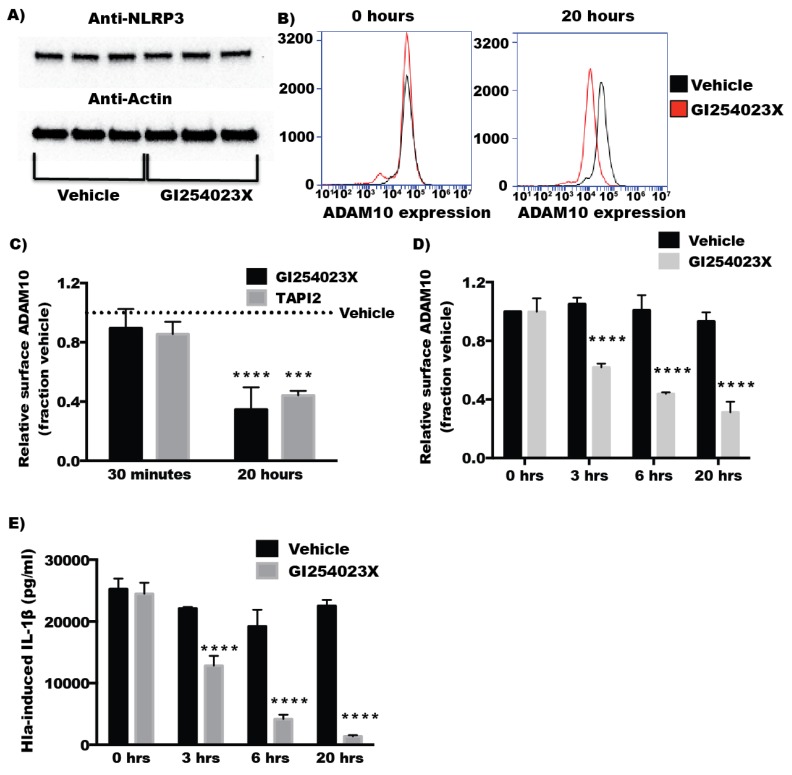
(**A**) Cells were treated with either vehicle (DMSO) or GI254023X for 20 h and cell lysates were analyzed by immunoblot for NLRP3 and Actin (loading control) as indicated. Lysates from three separate vehicle treated cell populations and three separate GI254023X-treated cell populations were tested. (**B**) Representative histograms showing the cell surface staining in vehicle- or ADAM10 inhibitor-treated THP1 cells at either 0 or 20 h of inhibitor treatment; (**C**) THP1 cells were treated with GI254023X, TAPI2, or vehicle overnight or 15–30 min before flow cytometric analysis of ADAM10 cell surface expression. Each bar represents the mean of the mean fluorescent intensity of cell surface staining from multiple experiments (*n* = 3) (**D**–**F**) THP1 cells were treated with GI254023X or DMSO for 0, 3, 6 h, or overnight before flow cytometric analysis of ADAM10 cell surface expression or treatment with α-hemolysin was carried out as above. (*n* = 3) For (**C**), *** and **** Indicates statistically significant reduction when compared to vehicle-treated controls (*p* ≤ 0.001 and *p* ≤ 0.0001, respectively) determined by one-way ANOVA with Dunnett’s multiple comparisons testing; for (**D**,**E**), **** Indicates statistically significant reduction when compared to vehicle treated control at the same time point (*p* ≤ 0.0001) determined by two-way ANOVA with Sadik’s multiple comparisons testing.

**Figure 6:**
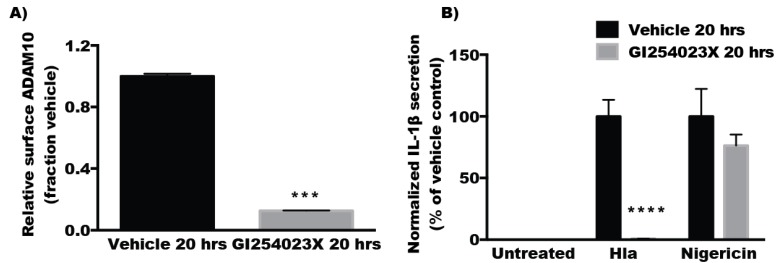
(**A**) U937 cells were treated with GI254023X or vehicle for 20 h and ADAM10 cell surface expression measured. (**B**) PMA-differentiated U937 cells were treated with GI254023X or vehicle for 20 h prior to treatment with α-hemolysin. For (**A**) *** Indicates statistically significant reduction when compared to vehicle treatment (*p* ≤ 0.0001) determined by paired *t*-test; for (**B**) **** Indicates statistically significant when compared to vehicle treated control cells (*p* ≤ 0.0001) determined by one-way ANOVA with Sadik’s multiple comparisons testing.

In contrast to the findings with short-term inhibition of ADAM10, overnight treatment with GI254023X diminished Hla-mediated but not nigericin-induced cytotoxicity in THP1 cells (Figure 4A). Overnight treatment with TAPI2, also caused a downward trend in Hla-induced death, though this did not meet statistical significance (Figure 4B). Overnight treatment with TAPI2 or GI254023X inhibited Hla-induced IL-1β secretion but not nigericin-induced IL-1β secretion in THP1 cells while 30 min inhibitor treatment had no effect on IL-1β secretion (Figure 4C–F) Because inhibition of ADAM10 protease activity did not immediately diminish Hla-induced NLRP3 inflammasome activation, we tested whether prolonged inhibitor treatment altered NLRP3 expression levels using immunoblot analysis. Levels of NLRP3 were not altered by treatment of THP1 cells with GI254023X for 20 h (Figure 5A), consistent with the continued response of these cells to nigericin (Figure 4D,F). We then sought to determine whether chemical inhibition altered the quantity of surface expressed ADAM10. Treatment of THP1 cells with either inhibitor for 30 min had no significant effect on cell surface expression of ADAM10 detectable by flow cytometry. After 20 h, treated THP1 exhibited diminished detectable surface ADAM10 (Figure 5A–C). To better understand the time dependent difference in the response of THP1 cells treated with these inhibitors, we assessed the ADAM10 cell surface expression over time during treatment with GI254023X using flow cytometry. Cell surface expression of ADAM10 decreased over time when compared to vehicle treated cells. As expected, the loss of ADAM10 expression was accompanied by similar reductions in Hla-induced IL-1β secretion at the corresponding timepoints in THP1 cells (Figure 5D,E). To ensure the effects of ADAM10 inhibition on ADAM10 cell surface expression and Hla-induced IL-1β was not limited to THP1 cells, we tested whether prolonged GI254023X exposure in U937 cells had an effect on ADAM10 levels and IL-1β secretion. U937 cells treated with GI254023X phenocopied effects observed with THP1 cells (Figure 6).

## 3. Experimental Section

### 3.1. siRNA Transfection of THP1 Cells

THP1 cells were purchased from ATCC (Manassas, VA, USA). THP-1 cells were maintained in RPMI 1640 media (Gibco. Thermo Fisher Scientific, Waltham, MA. USA) supplemented with 10% Fetal calf serum and Penicillin and Streptomycin as described in prior studies [15,26]. Cells were transfected with the TransIT-TKO^®^ transfection reagent from Mirus (Madison, WI, USA) per the manufacturers protocol (product #: MIR 2150). Cells were transfected with 50 nM of pooled siRNA constructs in 6 well plates. siRNAs were obtained from the GE/Dharmacon (Lafayette, CO, USA) siGenome library. A set of 4 siRNA was ordered for both ADAM10 (product #: D-004503-01-0002, D-004503-03-0002, D-004503-04-0002, D-004503-05-0002) and NLRP3 (product #: D-017367-01-0002, D-017367-02-0002, D-017367-03-0002, D-017367-04-0002) and pooled prior to transfection. Control siRNA used, include LaminA/C (product #: D-001050-01-05), and a pool of non-targeting siRNA (Non-targeting siRNA 2, 4, and 5) (#: D-001210-02, D-001210-04, D-001210-05), were ordered from GE/Dharmacon (Lafayette, CO, USA) as well. Cells were incubated for 3 days after transfection prior to use in subsequent experiments.

#### Immunotblot Analysis

Western blots were performed as done by Craven *et al.* [15]. Primary antibodies used were Adipogen Life Sciences (San Diego, CA, USA) anti NLRP3 monoclonal antibody (#: AG-20B-0014-C100) and ABCAM (Cambridge, MA, USA) anti-ADAM10 rabbit polyclonal (#: ab1997). Blots were imaged using a FluorChem E system from ProteinSimple (San Jose, CA, USA).

### 3.2. Treatment of Cells with Inhibitors

GI254023X was purchased from TOCRIS Bioscience (Bristol, UK) (product #: 3995) or Sigma (St. Louis, MO, USA, product #: SML0789) and resuspended in DMSO to either 10 mM or 20 mM stocks. TAPI2 was purchased from Enzo Life sciences (Farmingdale, NY, USA, product #: BML-PI135-0001) or Sigma (product #: SML04020, St. Louis, MO, USA) and resuspended in pyrogene free water to 10 mM. Cells were then treated with vehicle or inhibitor as indicated before to challenge with inflammasome activating stimuli.

### 3.3. Treatment of Cultured Cells with Inflammasome Activators for Cytokine Studies

THP-1 and U937 cells were suspended in RPMI 1640 media (Gibco, Thermo Fisher Scientific, Waltham, MA. USA) supplemented with 10% Fetal calf serum and Penicillin and Streptomycin at 1 × 10^6^ cells/mL and plated in tissue culture treated 24 or 48 well plates. U937 cells were treated overnight with phorbol 12-myristate 13-acetate (PMA) (Sigma, St. Louis, MO, USA product #: P1585). When indicated, the NLRP3 inflammasome was primed in the cells by treatment with *E. coli* lipopolysaccharide (Invivogen, San Diego, CA, USA, product #: tltl-3pelps) at a concentration of 100 ng/mL for 3 h. Recombinant α-hemolysin prepared as described by Craven *et al.* [15], α-hemolysin (30 μg/mL), Nigericin (50 μM) (Sigma, St. Louis, MO, USA product #: N7143) or ATP (100 mM) was added at the indicated concentrations to induce NLRP3 inflamamsome activation. After 1 h, cells and supernatants were collected by centrifugation at 17,000 ×*g* for 3 min. Cell culture supernatants were stored frozen at −80 °C until assayed for cytokine content. LDH activity was measured in supernatants using the Cyto-tox ONE kit (Promega, Madision, WI, USA, product #: G7891) read on a Perkin Elmer inspire plate reader. IL-1β was measured in cell culture supernatants using either Perkin Elmer Alphalisa (Product #: AL220C, Waltham, MA, USA) or BD bioscience (product #: 557953, San Jose, CA, USA) ELISA kits as per manufacturer protocols.

### 3.4. Treatment of Cultured Cells with Inflammasome Activator for Cell Death Studies

Cells were suspended in fresh media at 1 × 10^6^ cells/mL and plated in tissue culture treated 24 or 48 well plates. Recombinant α-hemolysin was prepared as described by Craven *et al.* [15]. Cells were treated with α-hemolysin (30 μg/mL) or Nigericin (50 μM) at the indicated concentrations to induce NLRP3 inflamamsome activation. After 1 h, Cells and supernatants were collected by centrifugation at 17,000× *g* for 3 min. Prior to the end of the experiment a control group of cells were lysed using 1% Triton X-100 as a lysis control. Cell culture supernatants were stored frozen at −80 °C until assayed for LDH production.

### 3.5. Propidium Iodide Studies

Cells were suspended in fresh media at 1 × 10^6^ cells/mL and plated in tissue culture treated 48 well plates at 300 μL per well. Propidium Iodide was added to cells 5 min prior NLRP3 activating stimuli. Cells were incubated for 1 h prior to cells being washed once with PBS and fixed using BD stabilizing fixative (product #: 338036, San Jose, CA, USA). Cells were then assayed by flow cytometry for Propidium iodide staining on a BD Accuri C6 flow cytometer (San Jose, CA, USA).

### 3.6. Measurement of Caspase-1 Activity in Treated Cells

Caspase-1 activity was measured using the FLICA^®^ 660 caspase-1 assay far-red fluorescence kit, (Product #: 9122) from ImmunoChemistry Technologies LLC (Bloomington, MN, USA). Cells were plated in a 48 well at 300 uL per well at 1 × 10^6^ cells/mL. The FLICA reagent was added and incubated with cells for 15 min prior to the addition of the indicated inflammasome activator. Cells were then incubated for an additional 30 min, transferred to 1.5 mL tubes, and washed twice with PBS. Cells were resuspended in 300 uL of PBS and 50 uL of supplied fixative. Accumulation of fluorescent caspase-1 inhibitor was assayed by flow cytometry on a BD Accuri C6 flow cytometer (San Jose, CA, USA).

### 3.7. Measurement of ADAM10 Cell Surface Immunofluorescence Staining Protocol

siRNA transfected cells were resuspended in 2% BSA in 1 × PBS with 0.1% sodium azide at a concentration of 1 × 10^7^ cells/mL. One-hundred microliters of cells were added into sterile tubes with 5 uL of either PE mouse IgG1, κ Isotype control Ab from Biolegend (San Diego, CA, USA) (product #: 400113) or PE anti-human CD156c (ADAM10) from Biolegend (product #: 352703). Cells were incubated with antibodies for 15–20 min in the dark at 4 °C. Cells were then twice washed with 2 mL of buffer and spun down at 350G for 5 min. Cells were resuspended in 500 uL of buffer prior to being analyzed by flow cytometry on a BD Accuri C6 flow cytometer (San Diego, CA, USA).

In the inhibitor time course, assays cells were resuspended in fresh RPMI media at a concentration of 1 × 10^6^ cells/mL. Cells were plated at 300 uL per well in a 48 well sterile tissue culture treated plate. Inhibitors and vehicle controls were added at the indicated times after which cells were washed with 500 uL of cell staining buffer from Biolegend^®^ (San Diego, CA, USA) (product #: 420201) and spun down at 2500 RPM for 3 min. Cells were resuspended in 100 uL of cell staining buffer prior to the addition of 5 uL of either PE mouse IgG1, κ Isotype control Ab from Biolegend (product #: 400113, San Diego, CA, USA) or PE anti-human CD156c (ADAM10) from Biolegend (product #: 352703, San Diego, CA, USA). The plate was incubated at 4 °C for 15–20 min in the dark; after which the cells were washed twice with 500 uL of cell staining buffer, spun down at 2500 RPM for 3 min and resuspended in a final volume of 300 uL prior to being analyzed by flow cytometry on a BD accuri C6 flow cytometer.

### 3.8. Measurement of Metalloprotease Activity in Cells Treated with TAPI2

Metalloprotease activity was measured using the Mca-P-L-A-Q-A-V-Dpa-R-S-S-S-R-NH_2_ Fluorogenic Peptide Substrate III from R & D systems (catalog #: ES003, Minneapolis, MN, USA). THP1 cells were resuspended at a concentration of 1 × 10^6^ cells/ml in 25 mM Tris buffer at a pH 8.0 as per manufacturers protocol. The assay was run in a 96 well plate in a 100-μL reaction with a final concentration of 10 μM of substrate per well. Reads were done every 2 min and TAPI2 100 μM or vehicle were added after 12 min and reads continued for 30 min.

### 3.9. Purified ADAM10 Activity Assay

This assay was conducted using the SensoLyte^®^ 520 ADAM10 Activity Assay from ANASPEC (Fremont, CA, USA) as per manufacturers protocol (Catalog #: 72226). GI254023X was used a final concentration of 20 μM and TAPI2 100 μM.

## 4. Conclusions

ADAM10 is important for α-hemolysin binding to target cells [9]. In addition, Hla binding leads to increased ADAM10 proteolytic activity in keratinocytes, endothelial cells, and epithelial cells [9,10,11,12]. This increased activity leads to disruption in cell-to-cell contacts through cleavage of E-cadherin and plays a key role in *S. aureus* pathogenesis. In addition to causing disruption of cell-to-cell contacts, Hla is known to induce potent pro-inflammatory signals in myeloid cells, including the production of IL-1β and induction of programmed necrotic cell death [15]. These pro-inflammatory actions of Hla require host cell NLRP3 inflammasome activity [15]. Targeted deletion of ADAM10 in myeloid cells results in diminished lung levels of IL-1β in a mouse model of *S. aureus* pneumonia [13]. In a *S. aureus* sepsis model, elimination of ADAM10 from platelets and myeloid lineages resulted in diminished IL-1β observed in liver homogenates, decreased lung and liver pathology, and decreased mortality in mice [14]. We have now shown that ADAM10 is required for Hla to activate the NLRP3 inflammasome in human monocytes (Figure 2). Our data suggest that the diminished tissue levels of IL-1β observed in these mouse models lacking ADAM10 are the direct result of diminished inflammasome activation in myeloid cells from the infected animals. Interestingly, loss of ADAM10 has also been implicated in lung epithelial injury from other bacterial pore-forming toxins, like pneumolysin from *Streptococcus pneumonia* [10]. We have demonstrated that myeloid cell ADAM10 is not required for NLRP3 activation by nigericin, a second pore-forming toxin. The difference in ADAM10 requirement for cellular injury between epithelial cells and monocytic cells in response to pore-forming toxins other than Hla may have to do with role of ADAM10 proteolytic activation in those cell types.

In mice, ADAM10 inhibitors prevent the loss epithelial integrity that is typically induced by *S. aureus* Hla during infections of the skin and lung [10,11]. The enhancement of ADAM10 proteolytic activity by Hla is clearly tied to that aspect of infection pathogenesis, which is likely important for the bacteria to establish invasive infection of the tissues the toxin is acting on. However, in published studies of *S. aureus* infection in mice lacking ADAM10 in myeloid lineage cells, the role of ADAM10 proteolytic activity, as opposed to high-affinity Hla binding, remains unknown. Our data now demonstrate that ADAM10 proteolytic activity is not required for host inflammasome activation in isolated immune cells. Because inhibitors of ADAM10 proteolytic activity ultimately result in reduced levels of myeloid cell surface expression of ADAM10, these inhibitors can still reduce Hla-induced inflammasome activation. These data suggest that in myeloid cells ADAM10-facilitated targeting of Hla to the cell is sufficient for NLRP3 inflammasome activation, which leads to both IL-1β secretion and cell death. The exact mechanism by which these inhibitors lower ADAM10 cell surface expression remains to be explored. ADAM10 is known to undergo autoproteolysis, it is possible that processing is important for maturation and/or proper trafficking of ADAM10 [27]. Although we have not demonstrated that pharmacologic ADAM10 inhibition reduces cell surface ADAM10 expression on other cell types, our findings open the possibility that ADAM10 inhibitors may improve outcomes in murine *S. aureus* infection models through multiple mechanisms including both reduction in ADAM10-mediated adherence factor cleavage and reduction of Hla-receptor on multiple cell types. Further, the mechanisms by which Hla activates NLRP3 remain to be elucidated but are also still potentially attractive therapeutic targets for adjunctive therapy to antibiotic therapy in severe *S. aureus* infections.

In mouse models, the consequences of the loss of Hla activity towards myeloid cells differs based on the location of infection. The loss of ADAM10 in the immune cell compartment or global loss of NLRP3 was beneficial to the host in a mouse pneumonia model of *S. aureus* infection, reducing mortality compared to mice with intact ADAM10 or NLRP3 [13]. Loss of ADAM10 from the myeloid lineage is deleterious to the host in the skin infection model of mouse staphylococcal disease by leading to increased lesion size and bacterial burden [13]. Prior studies by our group and others have tied Hla-induced inflammasome activation to worsened host outcomes in *S. aureus* infections [21]. Genetic deletion of NLRP3 improved clinical parameters in mouse pneumonia without effecting pathogen burden, suggesting that blunting the inflammasome mediated inflammatory response to *S. aureus* could be beneficial even after infection is established [21]. This combination of findings suggested that inhibition of ADAM10 could be an attractive mechanism to reduce deleterious effects of robust inflammatory response during severe *S. aureus* infection. Because the effects of Hla on myeloid derived human cells is likely redundant to several other pore-forming, immune cell-restricted toxins, known as leukotoxins, made by *S. aureus*, the role of Hla activity on immune cells in human infection is unknown. Like Hla, these leukotoxins also activate the NLRP3 inflammasome [26,28]. Thus, during *S. aureus* infection, the host inflammasome will be activated in immune cells regardless of the interaction between ADAM10 and Hla. The role of the NLRP3 inflammasome in mediating Hla effects on non-immune cells remains another open question in the field. Several studies have shown Hla antibody titers are important predictors of disease outcome in patients with *S. aureus* infections [29,30]. Gaining a better understanding of how Hla specifically contributes to human disease by targeting leukocytes and other cell types will be an important step in the development of novel, specific therapies for Staphylococcal disease.
